# Tumor-derived extracellular vesicles and genitourinary cancers: from biological mechanisms to clinical applications

**DOI:** 10.3389/fbioe.2025.1726884

**Published:** 2025-12-11

**Authors:** Xiaochui Wu, Xiaoyuan Xu, Jiancheng Du, Jianfeng Mei, Xiejuan Mao, Kecheng Lou

**Affiliations:** 1 Department of Urology, Lanxi People’s Hospital, Jinhua, Zhejiang, China; 2 Department of Intensive Care Unit, Lanxi People’s Hospital, Jinhua, Zhejiang, China; 3 Department of General Surgery, Lanxi People’s Hospital, Jinhua, Zhejiang, China

**Keywords:** extracellular vesicles, tumor-derived exosomes, genitourinary oncology, liquid biopsy, biomarkers, targeted therapy, precision medicine

## Abstract

**Background:**

Genitourinary cancers, including prostate, bladder, and kidney cancers, represent significant global health burdens. Their early diagnosis and effective treatment continue to pose substantial clinical challenges. Traditional diagnostic methods often suffer from invasiveness or insufficient accuracy, whereas liquid biopsy technologies—particularly the analysis of tumor-derived extracellular vesicles (TDEVs)—offer transformative potential for non-invasive diagnosis and precision medicine.

**Objectives:**

This review comprehensively examines the biological functions, diagnostic utility, therapeutic potential, current challenges, and future directions of TDEVs in genitourinary cancers, aiming to bridge the gap between mechanistic understanding and clinical translation.

**Methods:**

A systematic literature search was conducted across PubMed, Embase, and Web of Science databases to collect studies published between 2018 and 2024 on TDEVs in prostate, bladder, and kidney cancers, with a focus on molecular mechanisms, clinical applications, and technological advances. Following PRISMA guidelines, we established predefined inclusion and exclusion criteria, conducted dual independent screening of search results, and performed quality assessment of included studies. A narrative review approach was employed to synthesize the evidence.

**Key Findings:**

TDEVs exhibit a dual nature in genitourinary cancers: they function as “architects” of tumor progression by remodeling the tumor microenvironment, inducing metastasis, and conferring therapeutic resistance, while simultaneously serving as “multifunctional allies” in cancer treatment. Clinically, TDEV-based liquid biopsy markers demonstrate superior performance compared to conventional detection methods, with engineered TDEVs emerging as promising platforms for targeted drug delivery and immunotherapy. However, significant challenges remain in standardization of isolation protocols, characterization methods, and efficient targeting strategies.

**Conclusion:**

TDEVs represent a paradigm shift in precision oncology for genitourinary malignancies. With advancing technologies in isolation methods, multi-omics integration, and artificial intelligence applications, TDEVs are poised to become indispensable tools for early tumor detection, real-time monitoring, and personalized therapeutic strategies, heralding a new era in uro-oncological practice.

## 1 Introduction

Genitourinary malignancies (GU) constitute a substantial threat to global male health, encompassing diverse neoplasms such as prostate, bladder, and kidney cancers. Prostate cancer ranks as the second most common malignancy in men worldwide, with approximately 1.4 million new cases and 370,000 deaths documented in 2022 alone (Siegel et al., 2025). Bladder cancer occupies the 10th position in global cancer incidence and the 13th in mortality rates, exhibiting dramatically divergent 5-year survival rates—from 95.8% for non-invasive Ta stage to merely 4.6% for metastatic M1 disease (Antoni et al., 2017). Although kidney cancer presents with a relatively lower incidence rate, it is characterized by aggressive biological behavior, including early metastasis and frequent recurrence.

The current diagnostic and therapeutic frameworks for these malignancies face multiple unresolved challenges. In prostate cancer, despite prostate-specific antigen (PSA) screening improving detection rates, its insufficient specificity results in numerous false positives, with approximately 75% of men exhibiting elevated PSA levels harboring benign prostatic conditions upon biopsy (Wilkins et al., 2020; Catalona et al., 1999). For bladder cancer, the gold standard diagnostic method, cystoscopy, is associated with significant patient discomfort, while urine cytology demonstrates only 37% sensitivity for low-grade tumors (Babjuk et al., 2019). Kidney cancer, particularly in its early stages, lacks specific biomarkers, resulting in most patients being diagnosed at advanced, often incurable, stages.

These clinical dilemmas underscore the urgent need for non-invasive, highly specific diagnostic and monitoring tools. The emergence of liquid biopsy technologies offers a transformative approach to addressing this challenge, among which TDEVs have emerged as the most promising candidates due to their unique biological properties and molecular cargo.

Extracellular vesicles (EVs) are nano-sized (30–5,000 nm) membrane-bound particles secreted by nearly all cell types, classified into exosomes (30–150 nm), microvesicles (100–1,000 nm), and apoptotic bodies (500–5,000 nm) based on their biogenesis pathways, size, and biochemical composition (Théry et al., 2018). In this review, we use ‘extracellular vesicles (EVs)' as the general term for all membrane-bound vesicles released from cells, while ‘exosomes’ specifically refers to EVs originating from the endosomal pathway, and ‘microvesicles’ refers to EVs formed by direct outward budding from the plasma membrane. ‘TDEVs’ refers to EVs of tumor origin, encompassing all subtypes unless otherwise specified (Théry et al., 2018). This terminology follows the MISEV2018 guidelines recently updated in 2025 (Théry et al., 2018). TDEVs encapsulate a diverse array of bioactive molecules from their parent cells, including proteins, nucleic acids (mRNA, miRNA, lncRNA, circRNA), and lipids, enabling them to faithfully reflect the molecular characteristics and pathological state of tumors while maintaining remarkable stability in various body fluids (Whiteside, 2013).

Current research on TDEVs has generated both promising insights and significant controversies. While numerous studies highlight their potential as biomarkers and therapeutic vehicles, methodological challenges—including inconsistent isolation protocols, characterization techniques, and lack of standardized nomenclature—have limited the reproducibility and translation of findings (Théry et al., 2018; Wan et al., 2024). Furthermore, the functional heterogeneity of TDEVs may explain seemingly contradictory results regarding their roles in tumor progression (Di Vizio et al., 2012; Ramirez et al., 2018). This review aims to provide a balanced perspective on these issues, acknowledging both the tremendous potential and significant challenges in the field.

**FIGURE 1:**
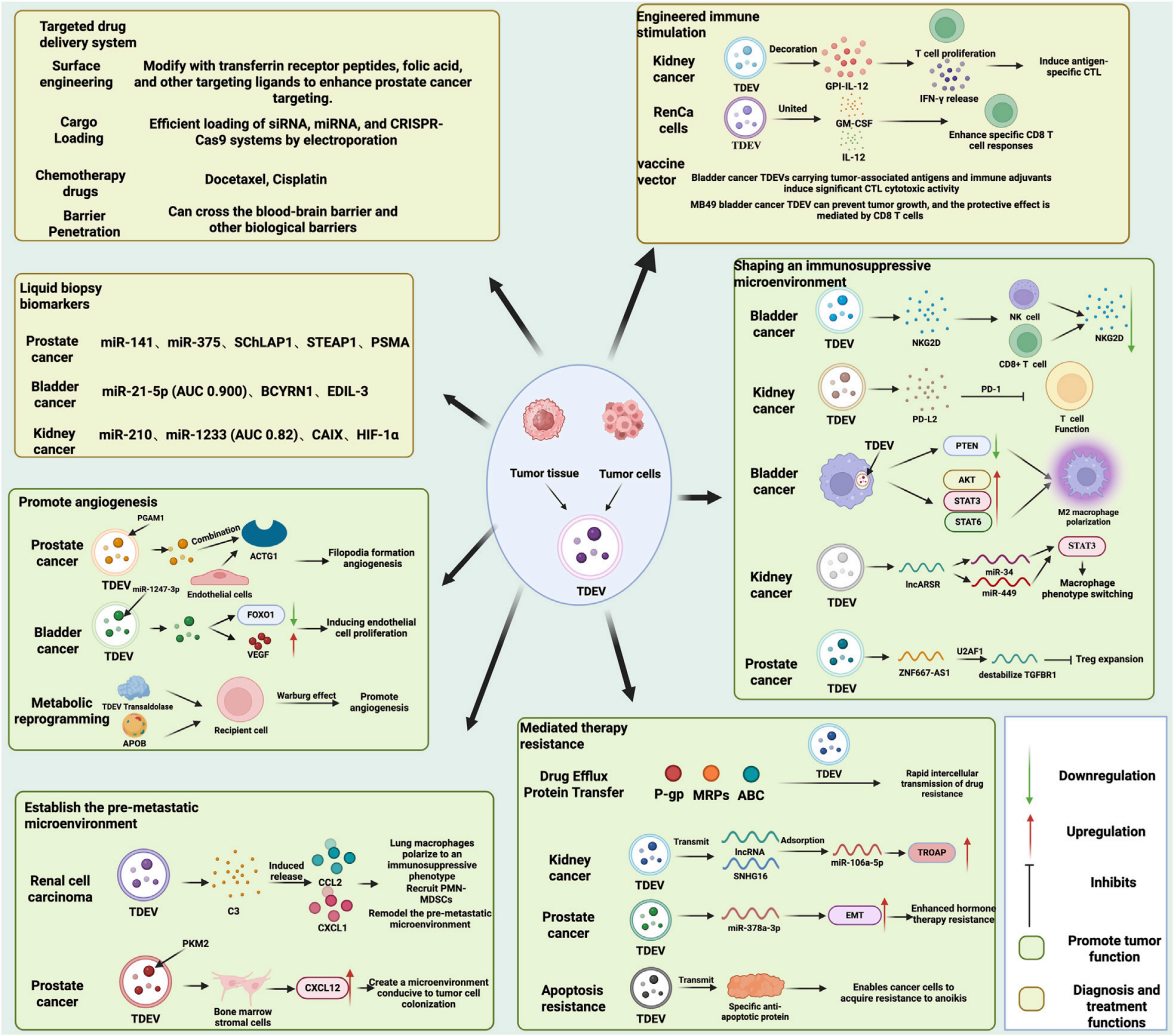
The dual roles and engineered applications of TDEVs in Urological Cancers.

This review systematically explores the dual nature of TDEVs in genitourinary cancers—both as drivers of tumor progression and as innovative diagnostic and therapeutic platforms—while critically evaluating current barriers and future opportunities for clinical translation (Figure 1). We critically examine conflicting evidence in the field, highlight methodological limitations that may explain discrepancies between studies, and offer original perspectives on addressing current challenges. Our comprehensive analysis aims to provide an authoritative reference for researchers and clinicians working to advance the field of genitourinary oncology through TDEV-based approaches.

## 2 Methods

A systematic literature search was conducted following the Preferred Reporting Items for Systematic Reviews and Meta-Analyses (PRISMA) guidelines. The search strategy included three electronic databases: PubMed, Embase, and Web of Science. The search period spanned from January 2018 to December 2024 to ensure the inclusion of recent advances in the field. The search terms included combinations of “extracellular vesicles” OR “exosomes” OR “microvesicles” OR “tumor-derived” AND “prostate cancer” OR “bladder cancer” OR “kidney cancer” OR “genitourinary cancer” OR “urological cancer”.

Inclusion criteria: (1) Studies focusing on TDEVs in prostate, bladder, or kidney cancers; (2) Studies published in English between 2018 and 2024; (3) Original research articles or reviews that provided data on molecular mechanisms, diagnostic utility, or therapeutic applications of TDEVs; (4) Studies with clear methodology and sample size ≥10.

Exclusion criteria: (1) Duplicate publications; (2) Studies without full text available; (3) Studies with unclear methodology or sample size <10; (4) Case reports, conference abstracts, or editorials without original data.

**FIGURE 2:**
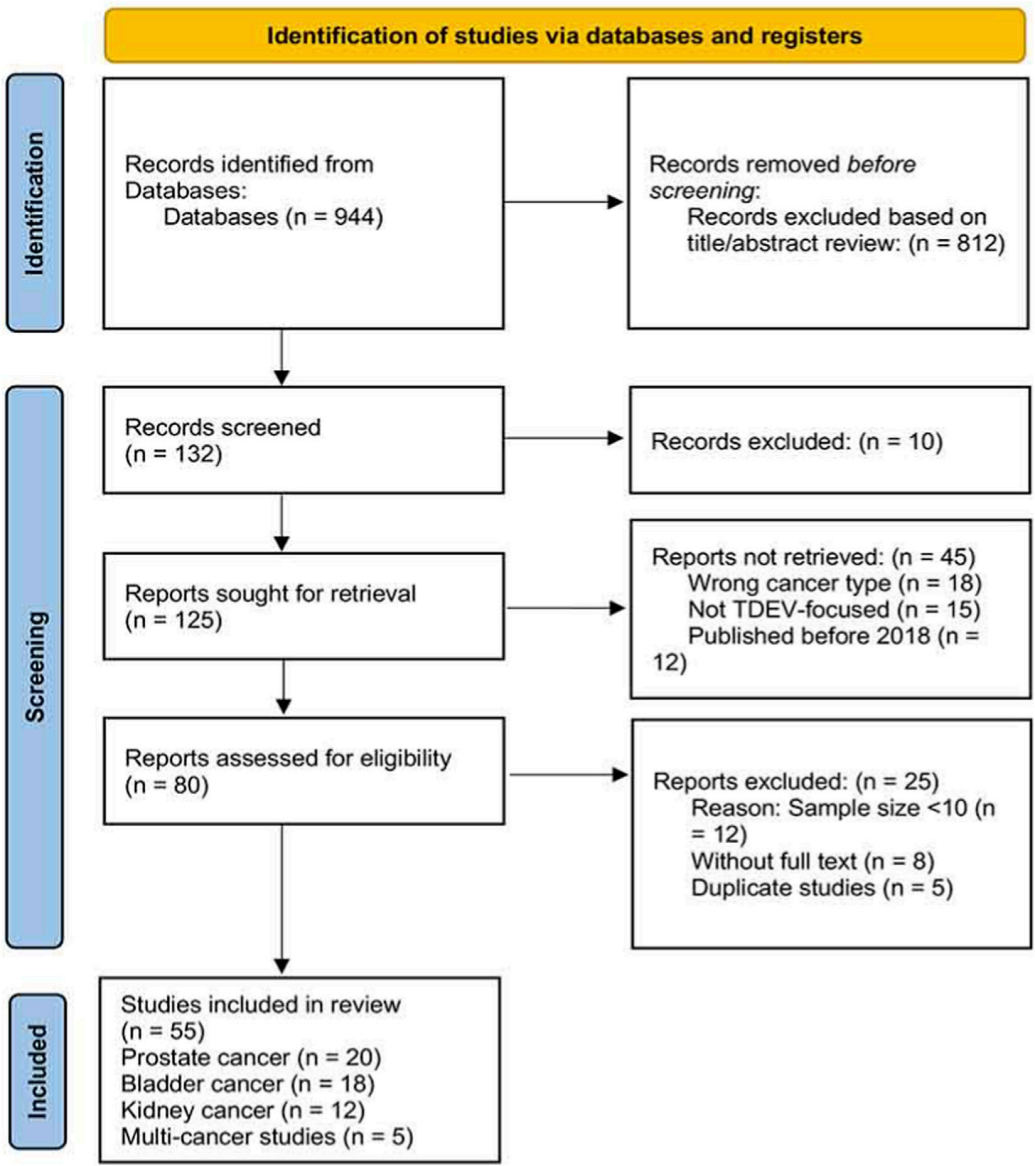
PRISMA flow diagram illustrating the study selection process.

Two reviewers independently screened titles and abstracts, with disagreements resolved through consensus or consultation with a third reviewer. Full-text articles were assessed for eligibility based on predefined criteria. The quality of included studies was evaluated using appropriate tools depending on study design. Data extraction included study characteristics, TDEV isolation methods, biomarker performance metrics, and clinical correlation data. The study selection process according to PRISMA guidelines is summarized in Figure 2.

## 3 Biological characteristics and classification of TDEVs

### 3.1 Biogenesis and release mechanisms of EVs

**FIGURE 3:**
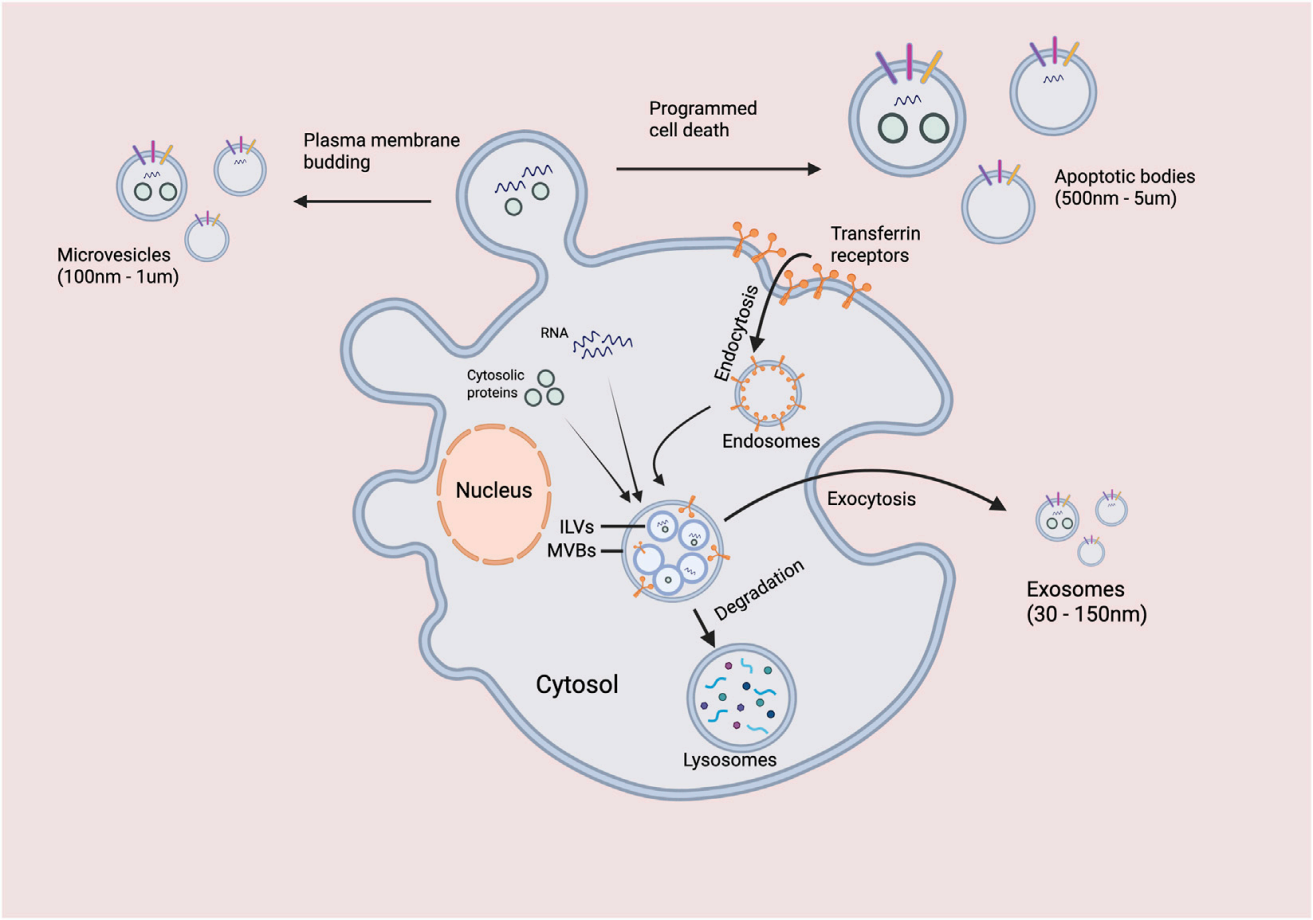
Schematic illustration of extracellular vesicle biogenesis pathways.

The biogenesis of EVs represents a sophisticated cellular process governed by multiple molecular mechanisms and intracellular signaling pathways, Figure 3. Based on their formation mechanisms, EVs are primarily classified into three categories:

Exosomes originate through the endosomal pathway, commencing with invagination of the plasma membrane to form early endosomes, which subsequently evolve into multivesicular bodies (MVBs) through inward budding of endosomal membranes (Pegtel and Gould, 2019). When MVBs fuse with the plasma membrane, their intraluminal vesicles are released as exosomes into the extracellular space (Colombo et al., 2014). This process is tightly regulated by the Rab GTPase family (particularly Rab27a and Rab27b) and the endosomal sorting complex required for transport (ESCRT). In tumor cells, specific oncogenic drivers (e.g., Ras, Src) can upregulate exosome release, while tumor suppressor genes (e.g., p53) typically inhibit this process (Hoshino et al., 2015).

Microvesicles, also known as ectosomes or shedding vesicles, are generated by direct outward budding from the plasma membrane, a process dependent on elevated intracellular Ca^2+^ concentrations and membrane phospholipid scrambling (Muralidharan-Chari et al., 2010). Enzymes such as phospholipase D2 and phospholipase A2 play crucial roles in microvesicle formation by facilitating membrane remodeling. Notably, tumor cells frequently exhibit dysregulated intracellular Ca^2+^ signaling due to metabolic abnormalities, leading to increased microvesicle release (van Niel et al., 2018).

Apoptotic bodies form from membrane blebbing during programmed cell death, typically ranging from 500–5,000 nm in size and containing various cellular components including organelles and nuclear fragments (György et al., 2011). In the tumor microenvironment, imbalances between apoptosis and proliferation can result in the aberrant release of apoptotic bodies, which may contribute to intercellular communication during disease progression.

The selective packaging of molecular cargo into EVs represents a highly regulated process influenced by the cellular origin and pathological state. Several biogenesis pathways govern this selective sorting: the ESCRT-dependent pathway, ESCRT-independent pathway involving lipid raft components, and the tetraspanin-rich microdomain pathway. In cancer cells, dysregulation of these biogenesis mechanisms—often driven by oncogenic mutations or tumor suppressor inactivation—results in EVs with unique molecular compositions that reflect the tumor’s malignant phenotype and therapeutic vulnerabilities (Hoshino et al., 2015; Larios et al., 2020; Wei et al., 2021).

This schematic illustrates the two primary pathways for the formation of extracellular vesicles. Microvesicles (100 nm - 1 µm) are generated by the direct outward budding and fission of the plasma membrane. In contrast, exosomes (30–150 nm) originate from the endosomal system: first, the plasma membrane invaginates to form early endosomes, which then mature into late endosomes. During this process, the endosomal membrane buds inward to form intraluminal vesicles (ILVs), creating a MVB. Cytosolic proteins and membrane receptors, such as transferrin receptors, can be incorporated into these ILVs. The MVB can subsequently follow one of two paths: (1) fusion with a lysosome, leading to the degradation of its contents, or (2) fusion with the plasma membrane (exocytosis), releasing the ILVs into the extracellular space as exosomes. Programmed cell death is also indicated as a potential contributing factor to vesicle release.

### 3.2 Molecular composition of TDEVs

The molecular cargo of TDEVs not only mirrors the molecular characteristics of their parent cells but is also selectively packaged and released under the regulation of complex biogenesis mechanisms. This selective packaging is controlled by multiple intracellular signaling pathways, including:

- ESCRT (Endosomal Sorting Complex Required for Transport)-dependent pathway: This constitutes the primary mechanism for exosome formation. The ESCRT complex (comprising ESCRT-0, -I, -II, and -III subunits) and associated proteins (such as TSG101, Alix) facilitate membrane invagination within MVBs and the packaging of intraluminal vesicles. The activity of this pathway directly influences the loading efficiency of proteins and nucleic acids (Hoshino et al., 2015; Larios et al., 2020).
- ESCRT-independent pathway: This pathway operates independently of the ESCRT complex, instead relying on membrane remodeling mediated by lipids (notably ceramide) or interactions between tetraspanin family proteins (CD63, CD9, CD81) and the plasma membrane to drive vesicle budding (Colombo et al., 2014; Wei et al., 2021).
- Rab GTPase family: As molecular switches, Rab proteins—particularly Rab27a/b and Rab35—regulate vesicle transport, anchoring, and membrane fusion processes, thereby controlling the targeted release of TDEVs (Colombo et al., 2014; Wei et al., 2021; Ostrowski et al., 2010). In tumor cells, activation of oncogenes (such as Ras, Src) or dysfunction of tumor suppressor genes (such as p53) frequently leads to significant alterations in TDEV release and cargo composition through modulation of these pathways.

Proteomic analysis of TDEVs reveals that their membrane surfaces display specific markers from parent cells, while their interiors contain functional proteins from the cytoplasm and nucleus. In genitourinary cancers, TDEVs overexpress disease-specific markers such as prostate-specific membrane antigen (PSMA) and STEAP1 in prostate cancer; EGFR and HER2 in bladder cancer; and carbonic anhydrase IX (CAIX) in kidney cancer (Jansen et al., 2009). Additionally, TDEVs contain diverse signaling molecules, including growth factors, transcription factors, and enzymes, which play pivotal roles in tumor progression.

Nucleic acid components represent particularly valuable cargo in TDEVs, encompassing various RNA species including mRNA, miRNA, lncRNA, and circRNA (Po et al., 2020). miRNA profiles in TDEVs exhibit strong associations with tumor type, stage, and molecular subtype, with these regulatory RNAs capable of modulating gene expression in recipient cells to promote cancer hallmarks such as proliferation, invasion, and immune evasion. Notably, the selective packaging of specific miRNAs into TDEVs appears to be a regulated process that fine-tunes intercellular communication within the tumor microenvironment (Po et al., 2020). Lipid components, including cholesterol, sphingolipids, and phospholipids, not only maintain vesicle structural integrity but also participate in signal transduction pathways. Certain lipids, particularly ceramide, play regulatory roles in TDEV biogenesis and function (Lee et al., 2024).

### 3.3 Isolation and characterization techniques for TDEVs

Accurate isolation and characterization of TDEVs represent fundamental prerequisites for studying their biological functions and clinical applications. Currently employed isolation methods include ultracentrifugation (UC) (Witwer et al., 2013), density gradient ultracentrifugation (DGUC) (Heijnen et al., 1999), size exclusion chromatography (SEC) (Leggio et al., 2023), polymer precipitation (Jong et al., 2017), immunoaffinity capture (Wang et al., 2021), and microfluidic technology (Hassanpour et al., 2021), each with distinct advantages and limitations (Wan et al., 2024) (Table 1).

**TABLE 1:** Critical comparison of TDEV isolation methods and impact on downstream applications.

Method	Key advantages	Major limitations	Critical impact on biomarker discovery	Critical impact on functional studies	Optimal downstream applications
Ultracentrifugation (UC)	High yield, simple setup	Co-pelleting contaminants, potential EV damage	May enrich contaminants, affecting biomarker specificity	Altered EV structure may impact functional characterization	Initial screening, low-budget studies
Density gradient centrifugation	High purity, separates subpopulations	Low yield, time-consuming, gradient contamination	Enables isolation of specific TDEV subpopulations with distinct biomarkers	Preserves EV integrity and functional properties	Studies requiring pure EV subpopulations
Size exclusion chromatography	Preserves EV integrity, no chemical damage	Low throughput, sample dilution	Maintains native EV surface markers, crucial for membrane biomarkers	Maintains cargo integrity for functional assays	Studies focusing on membrane proteins or delicate cargo
Polymer precipitation	Rapid, low cost	High contaminant levels, polymer interference	High false-positive rates due to polymer-protein complexes	Polymer residues may interfere with cell-based assays	Rapid screening in clinical settings
Immunoaffinity capture	High specificity for TDEV subtypes	Low yield, high cost, marker dependency	Enables detection of low-abundance TDEV-specific biomarkers	Selective isolation may bias functional representation	Targeted studies with known TDEV markers
Microfluidics	High efficiency, single-vesicle analysis	Limited scalability, complexity	Enables single-vesicle biomarker profiling	Preserves functional properties, enables single-vesule studies	Precision medicine applications, research

Notably, the choice of isolation method not only affects the yield and purity of TDEVs but also significantly impacts downstream analysis results and interpretation (Théry et al., 2018; Wan et al., 2024). For example, ultracentrifugation, though widely used, may lead to vesicle aggregation or disruption, altering structural and biological properties, thereby affecting functional experimental results. Density gradient ultracentrifugation can obtain intact vesicles and subpopulations, but its complex procedure and time consumption limit high-throughput applications. Polymer precipitation is rapid and simple but may introduce polymers that interfere with downstream mass spectrometry analysis. Immunoaffinity capture offers high specificity but may miss functionally important vesicle subpopulations that lack the target marker; antibodies might also interfere with downstream analyses. Microfluidics represents the latest advancement, enabling single-vesicle level analysis, but its technical complexity and equipment costs limit widespread application. Therefore, researchers must balance research objectives, sample characteristics, and technical feasibility when selecting isolation methods (Wan et al., 2024).

For comprehensive characterization, multiple analytical methods are required to assess the physical properties and molecular composition of TDEVs. These include nanoparticle tracking analysis (NTA) for determining concentration and size distribution, transmission electron microscopy (TEM) for morphological assessment, Western blotting for marker protein detection, and RNA sequencing for nucleic acid component analysis (Van Der Pol et al., 2010; Tulkens et al., 2020; Tiwari et al., 2021; Szatanek et al., 2017). Using orthogonal methods for combined characterization ensures the accuracy and reproducibility of research findings.

**FIGURE 4:**
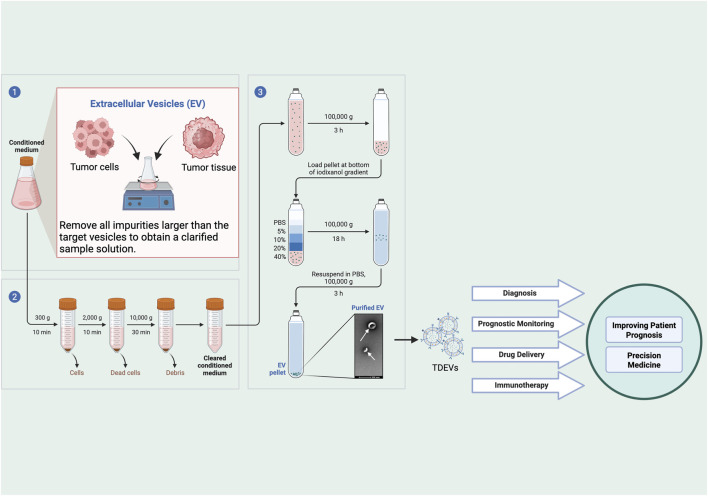
Workflow for the isolation, analysis, and clinical translation of TDEVs.

In technology selection, researchers must balance their research objectives with the limitations of various characterization methods. For example, although NTA can provide particle size distribution and concentration information, it is difficult to distinguish vesicle subpopulations with different densities; while TEM can directly visualize vesicle morphology, sample preparation may introduce artifacts; Western blotting typically requires large amounts of vesicle samples and has limited detection capability for low-abundance proteins; and RNA sequencing, although comprehensive for nucleic acid component analysis, may be contaminated by free RNA from cells. An integrated workflow encompassing TDEV isolation, purification, and clinical translation is depicted in Figure 4 (Van Der Pol et al., 2010; Tulkens et al., 2020; Tiwari et al., 2021; Szatanek et al., 2017).

## 4 The dual role of TDEVs in genitourinary cancers

### 4.1 TDEVs promoting tumor progression

TDEVs function as critical mediators of tumor development, coordinating progression, metastasis, and therapeutic resistance through multiple interconnected mechanisms that profoundly influence the tumor microenvironment. Their roles in promoting tumor progression are multifaceted, encompassing the stimulation of angiogenesis, metabolic reprogramming, induction of epithelial-mesenchymal transition (EMT), facilitation of anoikis resistance, establishment of pre-metastatic niches, and conferrence of therapy resistance.

Tumor angiogenesis is a prerequisite for growth and metastasis, a process effectively regulated by TDEVs (Wang et al., 2020a). For instance, prostate cancer-derived TDEVs are enriched with phosphoglycerate mutase 1 (PGAM1), which promotes filopodia formation and angiogenesis in human umbilical vein endothelial cells (HUVECs) by binding to γ-actin (ACTG1), thereby enhancing the invasive and metastatic capabilities of tumor cells (Luo et al., 2023). In bladder cancer, TDEVs carrying miR-1247-3p accelerate angiogenesis by inhibiting FOXO1 and enhancing VEGF secretion (Liu Z. et al., 2024). Beyond signaling molecules, TDEVs can directly transfer glycolytic enzymes and metabolites, such as apolipoprotein B-100 (APOB), to recipient cells, promoting the Warburg effect and sustaining tumor growth under nutrient-limited conditions (Lee et al., 2025). Cancer-associated fibroblast (CAF)-derived TDEVs play a particularly important role in this metabolic reprogramming by directly transferring functional metabolites, including amino acids, lipids, and tricarboxylic acid cycle intermediates, to cancer cells (Zhao et al., 2016).

The epithelial-mesenchymal transition (EMT) is a critical step in metastasis, and TDEVs are potent inducers of this process. Prostate cancer-derived TDEVs contain circFMN2, which, upon binding to HuR, reduces the HuR-KLF2 interaction, inhibits KLF2 expression, and subsequently decreases RNF128 transcription, collectively promoting tumor cell proliferation, invasion, and migration (Huang et al., 2023). In bladder cancer, the long non-coding RNA LINC00665 within TDEVs epigenetically upregulates RAB27B expression, forming a RAB27B-HGF-c-Met positive feedback loop that enhances lymphangiogenesis and lymph node metastasis (Li et al., 2023). Furthermore, TDEVs confer resistance to anoikis—the programmed cell death following detachment from the extracellular matrix—by delivering specific anti-apoptotic proteins and regulating survival signaling pathways, thereby facilitating the survival of circulating tumor cells (Di Vizio et al., 2012). TDEVs also actively remodel the extracellular matrix and modulate the immune microenvironment of distant tissues to create “pre-metastatic niches” that foster secondary tumor formation (Halin et al., 2016). A specific example in renal cell carcinoma involves complement C3 within TDEVs, which promotes polarization of lung macrophages toward an immunosuppressive phenotype and recruits polymorphonuclear myeloid-derived suppressor cells (PMN-MDSCs), thereby remodeling the pre-metastatic microenvironment to facilitate metastasis (Zhang et al., 2025).

Therapeutic resistance represents another critical mechanism by which TDEVs contribute to treatment failure. They can actively efflux chemotherapeutic drugs and horizontally transfer drug-resistance proteins such as P-glycoprotein (P-gp) and ABC transporters, with this resistance transfer occurring rapidly within hours (Yang et al., 2020). In sunitinib-resistant clear cell renal carcinoma, TDEVs transmit the long non-coding RNA SNHG16, which acts as a competing endogenous RNA (ceRNA) for miR-106a-5p, leading to TROAP protein upregulation and activation of downstream pro-proliferation pathways, thereby mediating resistance spread (Saimaiti et al., 2025). TDEVs also influence therapeutic sensitivity through modulation of autophagy and DNA repair pathways. For example, prostate cancer-derived TDEVs deliver miR-378a-3p, which promotes MAOA-mediated EMT and enhances resistance to hormone therapy (Wang et al., 2022).

A major challenge in leveraging TDEVs diagnostically and therapeutically lies in their high heterogeneity. Different TDEV subpopulations may originate from distinct tumor cell subclones or differentiation states, resulting in significant variations in molecular composition and function (Di Vizio et al., 2012; Ramirez et al., 2018). This heterogeneity impacts the reliability of biomarker detection and complicates the development of TDEV-based therapies. For instance, some TDEV subpopulations exhibit pro-metastatic properties, while others may demonstrate immune-activating functions (Ramirez et al., 2018). This functional diversity implies that future clinical applications may require the targeted capture and analysis of specific TDEV subpopulations. The development of single-vesicle analysis technologies, such as microfluidics and super-resolution microscopy, offers promising avenues to address this challenge, although their clinical translation still faces hurdles in standardization and reproducibility (Wang et al., 2020b; Beekman et al., 2019).

### 4.2 TDEVs promoting immune escape

Immune escape within the tumor microenvironment represents a hallmark of cancer progression, with TDEVs playing significant roles through multiple mechanisms that suppress host immune responses and foster immune tolerance.

TDEVs can inhibit T cell function through various pathways. First, TDEVs carry immunosuppressive molecules such as programmed death ligand 1 (PD-L1) and transforming growth factor-β (TGF-β), which directly inhibit T cell activation when delivered to immune cells (Liu T. et al., 2024). Studies have demonstrated that prostate cancer cell-derived TDEVs express NKG2D ligands on their surface, which downregulate NKG2D expression on NK cells and CD8^+^ T cells, thereby impairing their cytotoxic functions (Lundholm et al., 2014). Notably, surface NKG2D expression on circulating NK cells and CD8^+^ T cells in patients with castration-resistant prostate cancer (CRPC) is significantly lower than in healthy individuals, and incubating healthy lymphocytes with serum from CRPC patients can induce rapid downregulation of NKG2D expression on effector lymphocytes (Lucien et al., 2022).

In kidney cancer, exosomes derived from clear cell renal carcinoma cells primarily express PD-L2 rather than PD-L1 on their surface. Under immunocompetent conditions, these exosomal PD-L2 molecules are captured by immune cells in a PD-1-dependent manner, systemically suppressing T cell function—increasing regulatory T cell proportions while decreasing cytotoxic CD8^+^ T cell proportions (Zhang et al., 2022). This unique immune escape mechanism suggests novel therapeutic approaches targeting the PD-1/PD-L2 axis in kidney cancer.

TDEVs also promote tumor immune escape by inducing differentiation of immunosuppressive cells. Renal cancer cell-derived exosomes transmit long non-coding RNA lncARSR, which binds to miR-34/miR-449 to increase STAT3 expression, thereby inducing macrophage phenotype switching, altered cytokine release, and impaired phagocytic function (Shi et al., 2024). Clinical data indicate that high macrophage infiltration correlates with poor prognosis in kidney cancer patients, underscoring the importance of this mechanism in disease progression. Additionally, TDEVs can promote regulatory T cell (Treg) expansion, with ZNF667-AS1 in prostate cancer cell-derived TDEVs inhibiting PC cell growth and docetaxel resistance by destabilizing TGFBR1 mRNA in CD4^+^ T cells through interaction with U2AF1, thereby reducing TGFBR1 expression and weakening Treg expansion (Jiang et al., 2021). This complex immune regulatory network provides critical insights into understanding and potentially disrupting the tumor immune microenvironment.

TDEVs also create an immunosuppressive microenvironment through multiple pathways. Bladder cancer cell-derived exosomes can be phagocytosed by macrophages, promoting their polarization toward immunosuppressive M2 phenotype—a process mediated by downregulating PTEN and activating AKT/STAT3/6 signaling pathways (Zhang et al., 2010). In prostate cancer, TDEVs increase CXCL12 production in bone marrow stromal cells (BMSCs) by transmitting PKM2, creating a microenvironment conducive to tumor cell engraftment (Dai et al., 2019).

Notably, the immunomodulatory effects of TDEVs exhibit context-dependent duality. In environments lacking adaptive immunity, exosomal PD-L2 from kidney cancer cells has been paradoxically shown to inhibit tumor growth and metastasis, demonstrating that their effects are highly dependent on the host immune context (Zhang et al., 2022). This complexity underscores the challenges of developing TDEV-based immunotherapies and highlights the need for personalized approaches that consider the specific immunological characteristics of individual patients and tumor microenvironments.

### 4.3 TDEVs as therapeutic application vehicles

Despite their roles in promoting tumor progression, the unique biological characteristics of TDEVs—natural biocompatibility, low immunogenicity, efficient cellular uptake, and targeted delivery capabilities—make them promising vehicles for therapeutic applications.

In immunotherapy, TDEVs serve as natural immune modulators with several advantages. Studies have demonstrated that renal cell carcinoma cell-derived exosomes, genetically modified to express glycosylphosphatidylinositol-anchored IL-12 (GPI-IL-12), retain renal cancer-associated antigen G250 and exhibit enhanced immunogenicity, significantly promoting T cell proliferation and IFN-γ release while effectively inducing antigen-specific cytotoxic T lymphocytes (CTLs) (Beckham et al., 2014). Similarly, RenCa cell-derived exosomes combined with cytokines GM-CSF and IL-12 can significantly enhance specific CD8^+^ T cell responses, inhibiting tumor growth in animal models (Zhang et al., 2009).

Bladder cancer-derived exosomes also show potential as vaccine carriers. Immunogenic exosomes derived from mouse MB49 bladder cancer cells can prevent MB49 tumor growth in mice, with this protective effect mediated by CD8^+^ T cells (Mathew et al., 2020). Furthermore, the surface properties of TDEVs can be engineered to enhance antigen presentation and activate specific immune responses. These studies indicate that tumor-derived exosomes can be engineered into effective immunotherapeutic tools, offering new approaches for personalized cancer vaccine development and immunotherapy.

For targeted drug delivery, TDEVs present several advantages as drug delivery platforms. Their natural biocompatibility and low immunogenicity allow them to evade clearance by the reticuloendothelial system (Xia et al., 2024; Kim et al., 2020). Second, their lipid bilayer structure protects therapeutic cargo from rapid degradation in biological fluids. Third, they can cross various biological barriers—including the blood-brain barrier—providing delivery pathways for hard-to-treat tumors (Zhou et al., 2024). Through genetic engineering or chemical modification, receptor-targeting ligands (such as transferrin receptor peptides, folic acid) can be conjugated to the surface of TDEVs to enhance their specific delivery to target cells (Wang et al., 2025a). Cargo loading methods include electroporation, incubation, and sonoporation, with electroporation demonstrating the highest efficiency for loading siRNA, miRNA, and even CRISPR-Cas9 systems (Chen et al., 2018).

In gene therapy applications, TDEVs can stably transport nucleic molecules and serve as non-viral vectors for gene therapy (Guo et al., 2020). Studies have shown that TDEVs loaded with tumor suppressor genes (such as p53, PTEN) can effectively inhibit tumor cell growth (Wang et al., 2025b). Additionally, TDEVs can be used to deliver siRNA or shRNA to silence oncogene expression. For example, TDEVs loaded with Bcl-2-targeting siRNA can enhance the sensitivity of bladder cancer cells to chemotherapeutic drugs (Li et al., 2020).

In recent years, the combination of the CRISPR-Cas9 gene editing system with TDEVs has opened new avenues for precise gene therapy and genome engineering. Studies have demonstrated that loading the CRISPR-Cas9 system into TDEVs via electroporation can achieve precise editing of specific genes with significantly lower immune responses compared to viral vectors (Zarovni et al., 2015). This platform technology provides promising new approaches for treating genetic mutations underlying hereditary genitourinary cancers and for correcting driver mutations in sporadic cancers. Moreover, TDEVs can be engineered to carry multiple therapeutic payloads simultaneously—including siRNAs, chemotherapeutic drugs, and immune modulators—enabling combinatorial therapeutic strategies within a single delivery vehicle.

TDEVs play a dual role in genitourinary cancers: on one hand, they exert tumor-promoting functions by transferring specific molecules (e.g., PGAM1, miR-1247-3p, PD-L2, lncARSR) to promote angiogenesis, shape an immunosuppressive microenvironment, establish pre-metastatic niches, and mediate therapy resistance; on the other hand, they serve diagnostic and therapeutic functions by enabling liquid biopsy diagnostics (e.g., biomarkers such as miR-141 and miR-21-5p), achieving targeted drug delivery through surface engineering (loading chemotherapeutic agents/gene-editing systems), and functioning as engineered immune stimulators (e.g., GPI-IL-12-modified vaccine vectors), demonstrating their translational potential in precision medicine.

## 5 TDEVs as biomarkers for genitourinary cancers

### 5.1 Advantages and application value of liquid biopsy

Liquid biopsy has emerged as a transformative diagnostic approach that complements conventional tissue biopsy methods. As essential components of liquid biopsy, TDEVs offer several advantages over other circulating biomarkers, including high tumor specificity, dynamic monitoring capabilities, comprehensive molecular information, and ease of sample acquisition.

TDEVs contain tumor-specific molecular markers on their surfaces and within their structure, such as PSMA and STEAP1 in prostate cancer; EGFR and HER2 in bladder cancer; and CAIX in kidney cancer (Jansen et al., 2009). These membrane-bound markers enable more accurate identification of tumor origins and improve diagnostic specificity. Furthermore, TDEVs reflect the real-time status of tumors, with their molecular profiles changing dynamically in response to disease progression and treatment interventions (Li et al., 2016).

The molecular cargo of TDEVs provides multi-dimensional tumor information, including genomic variations, transcriptomic expression patterns, and post-translational protein modifications, thereby offering comprehensive data support for precision medicine (Han et al., 2022). Samples from body fluids such as blood, urine, and seminal fluid are relatively easy to collect with minimal patient discomfort, allowing for repeated sampling to facilitate treatment monitoring and prognosis assessment. This property is particularly valuable for patients who cannot undergo repeated tissue biopsies or for diseases like prostate cancer requiring long-term monitoring (Elsharkawi et al., 2019). Additionally, the stability of TDEV molecular cargo in stored samples makes them suitable for centralized testing and retrospective analysis in multi-center studies.

### 5.2 TDEV biomarkers in prostate cancer

Prostate cancer represents one of the genitourinary malignancies where TDEV biomarker research has been most extensively pursued, with numerous TDEV-derived biomarkers demonstrating promising diagnostic and prognostic value.

miRNAs constitute the most extensively studied nucleic acid biomarkers in TDEVs. For example, miR-141 is significantly overexpressed in serum exosomes of prostate cancer patients compared to those with benign prostatic hyperplasia (BPH) and healthy controls, with an area under the curve (AUC) of 0.869 for distinguishing metastatic from localized prostate cancer (Bhagirath et al., 2018); combined detection of miR-375 and miR-141 can further improve diagnostic accuracy (Bryant et al., 2012). Long non-coding RNAs such as SChLAP1 (Second Chromosome Locus Associated with Prostate-1) are highly expressed in prostate cancer TDEVs and correlate with tumor aggressiveness, effectively distinguishing between BPH and PCa when PSA levels fall within the diagnostic “gray zone” (4–10 ng/mL) (Wang et al., 2018).

Protein biomarkers offer complementary diagnostic value. STEAP1 (six-transmembrane epithelial antigen of the prostate 1), a prostate cancer-specific membrane antigen, demonstrates expression levels in TDEVs that correlate strongly with tumor burden. A multivariate model combining multiparametric MRI, PSA density, and STEAP1-EV density significantly improves prediction of clinically significant prostate cancer (csPCa), achieving an AUC as high as 0.90 (Logozzi et al., 2017). Additionally, other prostate-specific proteins such as PSMA (prostate-specific membrane antigen), PMSA (prostate-specific membrane antigen), and PAP (prostatic acid phosphatase) in TDEVs also demonstrate good diagnostic performance (Bijnsdorp et al., 2013). Multiple biomarker combinations can substantially improve diagnostic accuracy; for example, the SORTER technique combining six miRNAs (including miR-141, miR-375, etc.) achieves 100% accuracy in distinguishing Pca from BPH and 90.6% accuracy in differentiating metastatic from non-metastatic Pca (Lei et al., 2023).

### 5.3 TDEV biomarkers in bladder cancer

TDEV biomarker research for bladder cancer has predominantly focused on urine samples, which directly contact tumor tissues and contain rich tumor-derived information. Urinary exosomal miRNAs represent the most extensively studied bladder cancer biomarkers.

For instance, miR-21-5p is significantly overexpressed in urine exosomes of bladder cancer patients, with diagnostic performance superior to urine cytology (AUC 0.900) (Liu Z. et al., 2024); miR-155-5p and miR-146a also demonstrate good diagnostic value and show associations with tumor stage and progression (Zheng et al., 2021). Long non-coding RNAs such as BCYRN1 (BCYRN1 non-coding RNA) are substantially upregulated in urine-derived exosomes of bladder cancer patients and correlate with lymph node metastasis and poor prognosis. Mechanistic studies indicate that BCYRN1 can promote lymphatic metastasis by activating the Wnt/β-catenin signaling pathway (Yin et al., 2025). Protein biomarkers including EDIL-3 (EGF-like repeats and discoidin I-like domains 3) are highly expressed in exosomes derived from high-grade bladder cancer cells and promote tumor migration by activating the EGFR signaling pathway, positioning EDIL-3 as a potential therapeutic target (Zhao et al., 2025). KPNA2 (karyopherin subunit alpha 2), as a nuclear import protein, shows increased expression in bladder cancer TDEVs and can induce fibroblast transformation into cancer-associated fibroblasts (CAFs), suggesting its utility as a novel tumor biomarker (Xue et al., 2017).

Combinations of multiple urinary exosome biomarkers can further improve diagnostic accuracy. For example, a diagnostic panel composed of three exosomal lncRNA biomarkers (G023016, RP11-553N19.1, and LINC0087) achieves an AUC of 0.809 in early diagnosis of non-muscle-invasive bladder cancer (NMIBC), significantly outperforming traditional urinary cytology (Deng et al., 2022).

### 5.4 TDEV biomarkers in kidney cancer

Research on TDEV biomarkers for kidney cancer, while relatively limited compared to prostate and bladder cancer, has identified several promising biomarkers with diagnostic and prognostic utility.

miR-210 is significantly upregulated in urine exosomes of kidney cancer patients and correlates strongly with tumor hypoxia status, serving as a valuable diagnostic biomarker (Grange et al., 2011); miR-1233 also demonstrates good diagnostic performance with an AUC reaching 0.82 (Grange et al., 2015). Long non-coding RNAs such as lncARSR are upregulated in sunitinib-resistant renal cell carcinoma, mediating resistance by competitively binding miR-34/miR-449 to promote AXL and c-MET expression (Qu et al., 2016). Protein biomarkers including carbonic anhydrase IX (CAIX), a specific marker for clear cell renal carcinoma, show expression levels in TDEVs that correlate with tumor burden and response to therapy (Mallouk et al., 2023); proteins such as HIF-1α and VEGF in TDEVs also exhibit expression patterns associated with tumor progression and angiogenesis (Beekman et al., 2019). Importantly, TDEVs contain tumor-derived DNA, including mutations and copy number variations. Studies have demonstrated that large extracellular vesicles (L-EVs) released by renal cancer cells are enriched with chromosomal DNA, including large fragments up to 2 million base pairs, faithfully reflecting genomic abnormalities of parent cells (Sabaté Del Río et al., 2022). This represents a significant advancement for non-invasive genomic profiling of kidney cancers.

### 5.5 Challenges and solutions in clinical translation

Despite their substantial diagnostic potential, the clinical translation of TDEVs as biomarkers faces multiple challenges that must be addressed for successful implementation. The quantitative performance metrics of key TDEV biomarkers across different genitourinary cancers are summarized in Table 2.

**TABLE 2:** Quantitative analysis of clinically significant TDEV biomarkers in genitourinary cancers.

Cancer type	Fluid source	Key biomarkers	Clinical application	Performance metrics (AUC)	Sample size	Study design
Prostate cancer	Blood, urine	miR-141, miR-375, SChLAP1	Diagnosis, prognosis, biopsy decision	AUC: 0.869–0.90; sensitivity: 83%–90%	n = 150–300	Case-control, prospective cohort
Prostate cancer	Blood	STEAP1, PSMA	Diagnosis, load assessment	AUC: 0.90 (combined with MRI); specificity: 85%	n = 200–400	Diagnostic accuracy study
Bladder cancer	Urine	miR-21-5p, miR-155-5p	Diagnosis, disease monitoring	AUC: 0.900; sensitivity: 87%	n = 120–250	Case-control, longitudinal
Bladder cancer	Blood	BCYRN1, G023016	Early diagnosis, prognosis	AUC: 0.809 (lncRNA panel); HR: 2.34	n = 100–200	Retrospective cohort
Kidney cancer	Blood, urine	miR-210, miR-1233	Diagnosis, treatment monitoring	AUC: 0.82; sensitivity: 79%	n = 80–150	Case-control
Kidney cancer	Blood	CAIX, HIF-1α	Subtype assessment, therapeutic target	AUC: 0.75 (combined panel); response prediction: 82%	n = 60–120	Prospective cohort

Low abundance constitutes a major obstacle, as tumor-derived EVs may constitute only 0.0001% of total EVs in mixed samples, with the vast majority originating from non-tumor cells such as platelets (Whiteside, 2013). Solutions include developing highly sensitive detection techniques and targeted enrichment methods; for example, electrochemical sensing strategies achieve a detection limit as low as 5 tdEVs/μL through dual selectivity and dual amplification, covering clinically relevant concentration ranges (Singh et al., 2022; Xu et al., 2020).

Standardization presents another critical challenge, with the lack of standardized operating procedures from sample collection to analysis leading to poor reproducibility of research results (Théry et al., 2018). Although the International Society for Extracellular Vesicles (ISEV) has published MISEV guidelines, their widespread adoption in clinical settings remains challenging (Witwer et al., 2021). Solutions include establishing standardized operating procedures, promoting guideline implementation, and emphasizing transparent reporting of methodologies. In data analysis, TDEV omics data are inherently complex and high-dimensional, requiring advanced bioinformatics tools for effective processing (Kim et al., 2020). Artificial intelligence and machine learning techniques can be successfully applied; “ChatExosome” and other AI systems have demonstrated the ability to process complex Raman spectral data, enabling efficient, non-invasive clinical diagnosis of TDEVs by learning associations between spectral features and disease states (Yang et al., 2025). Biomarker validation in multi-center, large-sample cohorts represents another essential step toward clinical utility (Lucien et al., 2022). Establishing international multicenter research networks can accelerate biomarker validation and clinical translation through collaborative data sharing and standardized analytical protocols.

## 6 Clinical applications and therapeutic strategies of TDEVs

### 6.1 Clinical translation of liquid biopsy

TDEV liquid biopsy technology is gradually transitioning from research laboratories to clinical practice, with broad application prospects in genitourinary oncology. Several key applications are emerging:

In early diagnosis, TDEV biomarkers offer unique advantages. Studies have shown that prostate cancer cells transfer pyruvate kinase M2 (PKM2) to bone marrow stromal cells (BMSCs) through exosomes, upregulating CXCL12 production to “educate” the bone marrow microenvironment and create pre-metastatic niches. PKM2 expression is increased in serum exosomes from patients with primary or metastatic prostate cancer and correlates strongly with metastatic progression (Dai et al., 2019), suggesting its utility as an early metastasis biomarker.

In monitoring treatment response, dynamic changes in TDEV profiles can provide real-time insights into treatment efficacy. In a study of oligometastatic castration-resistant prostate cancer (omCRPC) patients undergoing stereotactic ablative radiotherapy (SABR), high baseline prostate cancer-derived extracellular vesicle (PCEV) levels were associated with shorter time to distant recurrence (median 3.5 months vs. 6.6 months), while increased PCEV levels after treatment (peaking on day 7) correlated with prolonged overall survival (median 32.7 months vs. 27.6 months) (Isebia et al., 2022). These dynamic changes provide a rational basis for timely treatment adjustment and response assessment. For prognostic evaluation, TDEV biomarkers demonstrate significant predictive value. Long non-coding RNA BCYRN1 in urine-derived exosomes of bladder cancer patients positively correlates with shorter survival and has been identified as an independent poor prognostic factor (Zheng et al., 2021). Similarly, specific miRNA combinations in prostate cancer TDEVs show strong associations with patients’ progression-free survival and overall survival outcomes (Zavridou et al., 2021).

In personalized treatment guidance, TDEV-based molecular typing can inform individualized therapy selection. Studies have shown that patients with different molecular subtypes of prostate cancer exhibit varying responses to specific treatments, and TDEV molecular profiles can help identify patient subgroups most likely to benefit from particular therapies (Signore et al., 2021). Such precise molecular typing approaches may help avoid ineffective treatments and reduce associated adverse effects.

### 6.2 Therapeutic applications of engineered TDEVs

The development of novel therapeutic strategies utilizing engineered TDEVs represents a cutting-edge research area with significant translational potential, encompassing targeted drug delivery systems, immunomodulatory therapy, tumor vaccine development, and gene therapy vectors.

In targeted drug delivery systems, the tumor-specific targeting of TDEVs can be enhanced by genetically modifying surface proteins; for example, engineering TDEV surfaces with transferrin receptor peptides improves their targeting efficiency to prostate cancer cells (Chen et al., 2018). For cargo loading, electroporation technology enables efficient loading of chemotherapeutic drugs, siRNA, or even CRISPR-Cas9 systems while maintaining cargo activity (Tai et al., 2019). Alternative loading strategies including incubation, extrusion, and sonoporation offer advantages for different cargo types, with microfluidic approaches providing more uniform and scalable loading processes. Immunomodulatory therapies utilizing engineered TDEVs show particular promise. Studies demonstrate that renal cell carcinoma cell-derived exosomes modified with glycosylphosphatidylinositol-anchored IL-12 (GPI-IL-12) significantly promote T cell proliferation and IFN-γ release, effectively inducing antigen-specific CTL production (Beckham et al., 2014). Similarly, TDEVs loaded with immune checkpoint inhibitors (such as anti-PD-1 antibodies) can be directly delivered to the tumor microenvironment, increasing local drug concentration while reducing systemic toxicity (Xu et al., 2019).

Tumor vaccine development represents another promising application of engineered TDEVs. Bladder cancer cell-derived exosomes loaded with tumor-associated antigens and immune adjuvants have induced significant CTL cytotoxic activity in preclinical models (Ghasroldasht and Agarwal, 2025). Additionally, exosome vaccines can enhance tumor antigen presentation and activate specific immune responses (Yang et al., 2013), with personalized vaccine strategies based on a patient’s own TDEVs emerging as a potential novel treatment approach.

In gene therapy applications, engineered TDEVs serve as effective non-viral gene vectors. Studies have demonstrated that TDEVs loaded with tumor suppressor genes (such as p53, PTEN) can effectively inhibit tumor cell growth with substantially lower immune responses than viral vectors (Jiao et al., 2022). Furthermore, TDEVs can be used to deliver siRNA or miRNA to silence oncogene expression, thereby inhibiting tumor growth and overcoming chemotherapy resistance (Yao et al., 2020). Recent advances combining TDEVs with CRISPR-Cas9 gene editing systems have opened new avenues for precise gene correction in hereditary genitourinary cancers and for targeting driver mutations in sporadic cancers. The development of “designer exosomes” with engineered targeting ligands, therapeutic payloads, and stimulus-responsive release mechanisms represents the next frontier in TDEV-based therapeutics.

### 6.3 Challenges and solutions in clinical translation

Despite their considerable promise, TDEV therapeutic strategies face multiple technical, regulatory, and commercial challenges that must be addressed for successful clinical translation.

Scalable production represents a major technical hurdle for large-scale manufacturing of clinical-grade TDEVs. Solutions include developing scalable cell culture systems and optimizing isolation and purification processes (Di Vizio et al., 2012), with microfluidic technology and automated production platforms expected to improve production efficiency and product consistency. Quality control and standardization constitute critical challenges for TDEV therapeutic products.

Establishing comprehensive quality control systems covering multidimensional assessment of physical properties, molecular composition, and biological activity is essential (Kalluri, 2016). Regulatory guidelines specifically for TDEV-based therapeutics remain underdeveloped, with clear need for industry-wide standards that can be adopted by regulatory agencies worldwide. Delivery efficiency and targeting represent additional challenges, as despite engineering improvements, *in vivo* delivery efficiency remains limited. Solutions include developing novel targeting ligands with higher affinity and specificity, and optimizing delivery routes based on tumor location and type (Elsharkasy et al., 2020). For instance, local administration (such as intravesical instillation for bladder cancer) can significantly improve treatment outcomes while reducing systemic exposure and potential adverse effects. Immunogenicity, although generally low for TDEVs, may still trigger immune responses with repeated administration. Surface engineering strategies to reduce immunogenicity or developing “stealth” technologies can help mitigate this issue (Li et al., 2021).

Notably, several clinical trials based on TDEVs are advancing, providing valuable technological foundations and clinical insights for their potential application in urological oncology. For instance, a completed trial in China (NCT03905028) demonstrated the safety and therapeutic potential of umbilical cord mesenchymal stem cell-derived extracellular vesicles in treating acute respiratory distress syndrome, highlighting the possibility of engineered vesicles as drug delivery systems. Another U.S.-based study (NCT04160053) evaluated the feasibility of an off-the-shelf allogeneic cellular product for severe lung injury, underscoring the prospects of standardized and scalable production of therapeutic vesicles. Looking ahead, these technological advances offer promising directions for urological tumors: TDEVs could serve as targeted “biological missiles” for precise drug delivery to prostate or bladder cancer cells, enable standardized liquid biopsy for early detection and recurrence monitoring, and modulate the tumor microenvironment to enhance immunotherapy efficacy. However, key challenges such as standardized sample processing, analytical consistency, and validation through large-scale clinical trials must be addressed to fully realize the potential of TDEVs in transforming precision diagnosis and treatment for urological cancers.

### 6.4 Regulatory and approval challenges

As emerging biotherapeutic products, TDEVs face significant regulatory and commercialization hurdles that must be addressed for successful clinical translation. A primary challenge lies in the unresolved regulatory pathway for TDEV-based products. Regulatory agencies worldwide, such as the US FDA and the European EMA, have yet to establish a clear classification for extracellular vesicles, leaving their designation as drugs, biologics, medical devices, or gene therapy products uncertain. This ambiguity directly impacts approval pathways, regulatory requirements, and development timelines. While guidelines for related fields like cellular and gene therapy exist, a specific framework for extracellular vesicles is still under development, creating significant uncertainty for developers (Kalluri, 2016). Beyond regulatory clarity, the biological complexity of TDEVs poses exceptional difficulties for establishing comprehensive quality control and release standards. This necessitates multi-dimensional assessment of physical characteristics (e.g., size, concentration), biochemical composition (e.g., marker proteins, nucleic acids), and biological activity (e.g., targeting ability, therapeutic potency). Furthermore, achieving scalable production and ensuring stringent batch-to-batch consistency are critical hurdles for industrialization and post-marketing surveillance, as each batch must maintain highly consistent safety and efficacy profiles (Kalluri, 2016). Early and continuous communication with regulatory agencies to jointly develop scientifically rigorous evaluation guidelines is therefore a crucial strategy for successful translation.

From a commercial and industrialization perspective, the TDEV sector holds substantial economic potential due to the high global incidence of genitourinary cancers, yet it faces significant feasibility challenges. The field is characterized by high technical barriers stemming from complex isolation and engineering technologies, which, while challenging, can provide a competitive advantage for early-mover companies with established technological dominance and intellectual property. Achieving cost-effectiveness through the development of automated, high-throughput production systems and the optimization of isolation and purification processes to achieve economies of scale is imperative for commercial success (Théry et al., 2018). Finally, payer acceptance and reimbursement decisions by healthcare systems will be decisive, underscoring the need for robust clinical and economic evidence to demonstrate superior value over existing methods and to secure favorable coverage decisions through innovative models such as value-based pricing or risk-sharing agreements.

### 6.5 Industrial prospects and economic considerations

The TDEV-related biotechnology sector holds substantial economic potential but also faces significant feasibility challenges that must be addressed for sustainable development.

The high global incidence of genitourinary cancers such as prostate cancer, bladder cancer, and kidney cancer renders the TDEV diagnostic market particularly attractive and clinically relevant. Technical barriers including complex isolation, analysis, and engineering technologies create high entry barriers that, while challenging, can provide competitive advantages for early-mover companies capable of establishing technological dominance and intellectual property protection. Cost-effectiveness considerations are paramount, with reducing production costs representing a key imperative for commercial success. Solutions include developing automated, high-throughput production systems and optimizing isolation and purification processes to achieve economies of scale (Théry et al., 2018). As applications expand and manufacturing processes mature, unit costs are expected to decrease substantially, improving accessibility and adoption. Payer acceptance constitutes another critical factor, as reimbursement decisions by healthcare systems will ultimately determine commercial success. Generating robust clinical and economic evidence demonstrating superior value compared to existing methods is essential for achieving favorable coverage decisions (Kim et al., 2020). Innovative payment models such as risk-sharing agreements and value-based pricing may improve payer acceptance by aligning reimbursement with clinical outcomes.

The schematic outlines the multi-step process for isolating and purifying EVs and their potential uses in oncology. The process begins with collecting conditioned medium from tumor cells or tumor tissues. This medium is then clarified by removing impurities such as cells and debris to obtain a cleared solution containing EVs. The EVs are pelleted via ultracentrifugation. For further purification, the EV pellet is resuspended and loaded into an iodixanol density gradient (e.g., 5%, 10%, 20%, 40%) and centrifuged. The purified EVs, which band at their characteristic density, are collected, washed in PBS, and pelleted again to yield a final, purified EV sample. These isolated TDEVs have significant clinical potential, including applications in diagnosis, prognostic monitoring, drug delivery, and immunotherapy. Ultimately, the utilization of TDEVs contributes to improving patient prognosis and advancing the field of precision medicine.

## 7 Future directions and prospects

### 7.1 Technological innovation and breakthroughs

Single vesicle analysis technologies represent a transformative future direction in TDEV research. Traditional bulk analysis approaches mask the inherent heterogeneity of TDEV populations, while emerging technologies such as microfluidics and super-resolution microscopy enable real-time imaging, molecular analysis, and functional assessment of individual EVs (Wang et al., 2020b; Beekman et al., 2019; Zhou et al., 2024). For example, EV-Ident technology has successfully identified highly efficient EV subpopulations with diagnostic and therapeutic potential through size fractionation and single vesicle analysis (Kim et al., 2020).

Integration of artificial intelligence and multi-omics approaches will be essential for extracting meaningful information from complex TDEV datasets. Machine learning algorithms can analyze vast datasets from TDEV proteomics, transcriptomics, lipidomics, and metabolomics to identify predictive biomarker signatures and establish molecular classifications (Greenberg et al., 2023). AI systems such as “ChatExosome” have demonstrated the ability to process complex Raman spectral data, achieving efficient, non-invasive clinical diagnosis of TDEVs by learning associations between spectral features and disease states (Yang et al., 2025). Novel separation and detection technologies will address limitations of traditional methods. Orthogonal barcoding-based smart nanodevices can achieve efficient isolation and proteomic analysis of TDEVs with minimal sample requirements (Wang et al., 2025a), while low-cost, portable detection platforms such as paper-based analytical devices (3D PADs) will enable point-of-care applications and resource-limited settings (Wang et al., 2025b). The development of *in vivo* real-time imaging technologies will be critical for understanding the pharmacokinetics and biodistribution of TDEVs in living organisms (Li et al., 2025; Zhang et al., 2020). Novel fluorescent probes and advanced imaging modalities such as two-photon microscopy may enable dynamic monitoring of TDEV behavior *in vivo*, accelerating mechanism research and therapeutic application development.

### 7.2 Deepening basic research

In-depth understanding of TDEV biogenesis and regulatory mechanisms may lead to the development of targeted intervention strategies. For example, modulating the activity of Rab GTPase family members or ESCRT complex components could affect TDEV release and consequently regulate tumor progression, with these findings potentially yielding novel therapeutic targets.

The complex interaction mechanisms between TDEVs and the tumor microenvironment require further elucidation. Research on how TDEVs remodel the immune microenvironment, angiogenic processes, and extracellular matrix components will enhance our understanding of tumor progression mechanisms (Clancy and D'Souza-Schorey, 2023) and guide the development of novel therapeutic strategies targeting TDEV-mediated communication. The heterogeneity of TDEVs represents the foundation of their functional diversity, as different TDEV subpopulations may exhibit specialized functions affecting specific biological processes (Di Vizio et al., 2012). Single vesicle analysis technologies will be instrumental in revealing the molecular basis of this heterogeneity and identifying functionally distinct subpopulations. Beyond their established roles, TDEVs may possess undiscovered functions in intercellular communication, such as in neuron-tumor crosstalk or systemic metabolic regulation (Kalluri, 2016). Maintaining an open scientific mindset and exploring unconventional research directions will expand the horizons of TDEV biology and potentially reveal novel clinical applications.

### 7.3 Clinical translation pathways

Establishing standardized systems for TDEV research and clinical application represents a priority for the field. Widespread adoption of ISEV guidelines and development of industry-specific standards and quality control specifications will be essential (Witwer et al., 2021). International collaborative initiatives such as a global TDEV research alliance could accelerate this process by harmonizing research protocols and facilitating data sharing.

Biomarker validation in multi-center, large-sample cohorts represents a critical step toward clinical implementation. Establishing international multicenter research networks to jointly advance biomarker validation and clinical translation will generate more robust evidence than single-center studies (Kim et al., 2020). Integrating TDEV detection with therapeutic drug development to develop companion diagnostics represents another promising approach. For example, screening patients suitable for immune checkpoint inhibitors based on their TDEV molecular profiles could improve treatment response rates and reduce unnecessary toxicity (Wang et al., 2020b). Such precision medicine models will enhance therapeutic efficiency and value. Developing individualized treatment plans based on patients’ TDEV molecular characteristics, integrating multi-omics data to establish predictive models guiding treatment selection, represents the future of personalized oncology (Zhou et al., 2024). This individualized approach will require sophisticated bioinformatics platforms and clinical decision support systems to effectively translate complex molecular data into actionable treatment recommendations.

### 7.4 Ethical and regulatory considerations

TDEV research raises several ethical and regulatory questions that require careful consideration as the field advances. Patient privacy and data security represent paramount concerns, particularly given the potential for TDEV analysis to reveal sensitive health information.

Ethical frameworks and robust data protection measures must be established, especially when TDEVs are used for risk assessment or health monitoring purposes. Informed consent processes need to adequately disclose the nature of TDEV testing and how results might be used. Novel regulatory challenges will arise as TDEV-based products and detection methods advance to clinical application. Regulatory agencies will need to update guidelines to accommodate these innovative technologies and products, potentially establishing a tiered regulatory framework that balances innovation promotion with patient safety protection. Ensuring equitable access to TDEV technologies and therapies represents another important ethical consideration. Technical complexity and high development costs may limit accessibility, particularly in resource-limited settings. Efforts should focus on developing simplified technologies and reducing costs, while considering approaches to ensure fair global access under principles of health equity. The long-term impacts of TDEV detection and treatment require careful assessment through well-designed long-term follow-up studies to monitor safety, efficacy, and socioeconomic impacts, with these data supporting continuous improvement of technologies and products.

### 7.5 Interdisciplinary collaboration and ecosystem building

TDEV research by nature requires multidisciplinary collaboration, with joint participation of biologists, clinicians, engineers, and computer scientists accelerating innovation and translation (Ramirez et al., 2018). For example, collaboration between microfluidic engineers and clinical researchers can develop practically applicable diagnostic tools that address real-world clinical needs.

Cultivating interdisciplinary TDEV research talent represents a long-term investment for the field. Establishing dedicated training programs and educational courses can develop researchers who understand both basic science and clinical applications, with early career development support crucial for attracting and retaining talented scientists in this emerging field. Promoting open sharing of data and methodologies through establishment of open-access databases and collaboration platforms will accelerate knowledge dissemination and application translation, while avoiding redundant research efforts. Academic-industry partnerships that combine fundamental research strengths with industrial development capabilities and regulatory expertise will be essential for successfully translating TDEV technologies from bench to bedside. Innovative collaboration models such as knowledge sharing agreements and pre-competitive consortia can foster win-win outcomes while maintaining appropriate intellectual property protection to incentivize continued investment and innovation.

## 8 Conclusion

This review uniquely synthesizes the role of TDEVs in genitourinary cancers by framing them through their fundamental duality: they are both architects of disease progression and multifunctional tools for clinical innovation. This perspective not only consolidates recent advances but also offers a unified framework for understanding the conversion of TDEVs from tumor accomplices into therapeutic allies.

In conclusion, this review systematically elucidates the complex roles of TDEVs in genitourinary cancers and their emerging clinical applications. TDEVs exhibit a fascinating dual nature in tumor progression: they function as “architects” promoting cancer development by remodeling the tumor microenvironment, inducing metastasis, and conferring therapeutic resistance; simultaneously, they serve as “multifunctional tools” for therapeutic applications, offering innovative platforms for diagnosis and treatment.

At the basic research level, TDEVs contribute to genitourinary cancer progression through various mechanisms, including stimulating angiogenesis, facilitating metastatic dissemination, mediating therapeutic resistance, and suppressing immune responses. These insights not only deepen our understanding of fundamental tumor biology but also provide rational targets for developing novel therapeutic strategies.

In clinical applications, TDEV-based liquid biopsy biomarkers demonstrate enormous potential, with their nucleic acid and protein cargo exhibiting excellent performance in diagnosis, prognosis assessment, and treatment monitoring across prostate, bladder, and kidney cancers. Engineered TDEVs also show distinctive advantages in targeted drug delivery, immunotherapy, and vaccine development, positioning them as next-generation therapeutic platforms.

Despite this promising outlook, TDEV research and clinical translation remain hampered by multiple challenges, including standardization of isolation and characterization methods, targeting efficiency limitations, clinical validation requirements, and industrialization barriers. Addressing these challenges will require sustained technological innovation, deeper fundamental research, well-defined clinical translation pathways, and comprehensive ethical and regulatory frameworks.

Looking ahead, TDEV research will advance along several interconnected trajectories: first, technological innovations promoting single vesicle analysis and AI applications; second, deepening basic research to understand TDEV biological functions and regulatory mechanisms; third, accelerating clinical translation through standardization initiatives and personalized therapy approaches; fourth, fostering interdisciplinary collaboration to drive technological breakthroughs and application innovation.

Furthermore, we fully acknowledge the importance of quantitative meta-analysis in establishing robust clinical evidence. Given the limitations of current studies, a key future endeavor will be conducting large-scale, multicenter multi-omics meta-analyses to precisely quantify the association between TDEV molecular cargo and patient prognosis across different urogenital tumors. This will provide a more solid, quantifiable basis for the application of TDEV in precision medicine.

With continued development in these areas, TDEVs are poised to achieve successful translation from laboratory research to clinical practice, becoming indispensable tools for precision diagnosis and treatment of genitourinary cancers. This evolution promises to bring patients more accurate diagnostic assessments and more effective therapeutic options, while providing novel conceptual frameworks and technical foundations for advancing precision oncology in urological malignancies.

However, as TDEV technology rapidly advances, we must carefully consider related ethical and societal implications. The widespread application of TDEVs raises a series of ethical issues, including patient privacy protection, data security, equitable access, and long-term monitoring. For example, TDEV analysis may reveal sensitive genetic information, necessitating the establishment of rigorous ethical frameworks and privacy protection measures. Furthermore, high development costs and complexity may lead to unequal distribution of medical resources, requiring policies to ensure fair access to technologies. Regulatory agencies need to establish clear guidelines that balance innovation promotion with patient safety protection. Establishing multi-stakeholder dialogue mechanisms, including scientists, clinicians, ethicists, patient representatives, and regulatory agencies, is crucial for ensuring the responsible development and application of TDEV technology.

## Data Availability

The original contributions presented in the study are included in the article/supplementary material, further inquiries can be directed to the corresponding author.
